# Transcriptomes Reveal Genetic Signatures Underlying Physiological Variations Imposed by Different Fermentation Conditions in *Lactobacillus plantarum*

**DOI:** 10.1371/journal.pone.0038720

**Published:** 2012-07-03

**Authors:** Peter A. Bron, Michiel Wels, Roger S. Bongers, Hermien van Bokhorst-van de Veen, Anne Wiersma, Lex Overmars, Maria L. Marco, Michiel Kleerebezem

**Affiliations:** 1 TI Food and Nutrition, Wageningen, The Netherlands; 2 NIZO food research, Ede, The Netherlands; 3 Kluyver Centre for Genomics of Industrial Fermentation, Delft, The Netherlands; 4 Centre for Molecular and Biomolecular Informatics, Radboud University Medical Centre, Nijmegen, The Netherlands; 5 Laboratory of Microbiology, Wageningen University and Research Centre, Wageningen, The Netherlands; 6 Host-Microbe Interactomics, Wageningen University and Research Centre, Wageningen, The Netherlands; University of Groningen, Netherlands

## Abstract

Lactic acid bacteria (LAB) are utilized widely for the fermentation of foods. In the current post-genomic era, tools have been developed that explore genetic diversity among LAB strains aiming to link these variations to differential phenotypes observed in the strains investigated. However, these genotype-phenotype matching approaches fail to assess the role of conserved genes in the determination of physiological characteristics of cultures by environmental conditions. This manuscript describes a complementary approach in which *Lactobacillus plantarum* WCFS1 was fermented under a variety of conditions that differ in temperature, pH, as well as NaCl, amino acid, and O_2_ levels. Samples derived from these fermentations were analyzed by full-genome transcriptomics, paralleled by the assessment of physiological characteristics, e.g., maximum growth rate, yield, and organic acid profiles. A data-storage and -mining suite designated FermDB was constructed and exploited to identify correlations between fermentation conditions and industrially relevant physiological characteristics of *L. plantarum*, as well as the associated transcriptome signatures. Finally, integration of the specific fermentation variables with the transcriptomes enabled the reconstruction of the gene-regulatory networks involved. The fermentation-genomics platform presented here is a valuable complementary approach to earlier described genotype-phenotype matching strategies which allows the identification of transcriptome signatures underlying physiological variations imposed by different fermentation conditions.

## Introduction

Lactic acid bacteria (LAB) are utilized as starter cultures in food fermentation for their spoilage-preventing and preservative effects on raw-materials, as well as their contribution to flavor and texture of the fermented product [1], [2], [3]. The wide industrial application of LAB supports the ambition to better understand and improve their fermentation characteristics. These include maximization of basic physiological variables such as growth rate and biomass production to increase fermentation efficiency [4], [5], the enhanced formation of desired metabolites and flavor profiles to optimize taste of the fermentation end-products [2], [3], [6], [7], as well as optimization of survival during industrial and downstream processing [5], [8].

The current public databases contain the complete genomes of over 30 LAB species (http://www.ncbi.nlm.nih.gov/genomes/lproks.cgi), while genome sequences of multiple strains are available for specific species [9], [10], [11]. This rapidly increasing genetic database of this group of bacteria enables in depth comparative genomics among strains of a species, or between different LAB species. Genomic diversity among strains of specific LAB species has also been assessed by DNA micro array-based comparative genome hybridization (CGH) approaches, providing one-directional gene absence-presence patterns [12], [13], [14], [15]. Such datasets in combination with random forest (RF)-based correlation [16] analysis, linking multiple-genome or CGH data to differential functional characteristics (phenotypes), has led to the identification of the genetic determinants important for some strain-specific phenotypes. For example, application of this strategy to *Lactobacillus plantarum* WCFS1 [9] revealed its mannose-specific adhesin-encoding gene [17] as well as several genetic loci involved in the immunomodulating capacity of this strain [13], [18].

Despite the successful examples of genotype-phenotype matching strategies, this approach intrinsically relies on diversity among strains at the level of their genome-content. Consequently, the importance of differential regulation of phenotypic characteristics that rely on expression of conserved genes cannot be assessed [8]. This limitation is clearly illustrated by the observation that closely related *Lactococcus lactis* strains display distinct expression patterns of conserved enzyme functions as a consequence of strain-specific regulation of gene expression [19] **.** Moreover, there is accumulating evidence that fermentation conditions may dramatically affect functional characteristics of LAB strains [20], [21]. These findings are supporting a complementary approach in which a specific LAB strain is grown under different fermentation conditions to induce phenotypic variation, followed by RF-based correlation of phenotypes and transcriptome profiles to pinpoint the genetic determinants responsible for the observed phenotypes (Figur 1). In addition, this approach also enables the identification of genes important for basal physiological parameters such as growth rate, which is not feasible with genotype-phenotype matching strategies, as variations in these parameters are relatively small for different strains of the same species when they are grown in rich laboratory media [8], [18].

**Figure 1:**
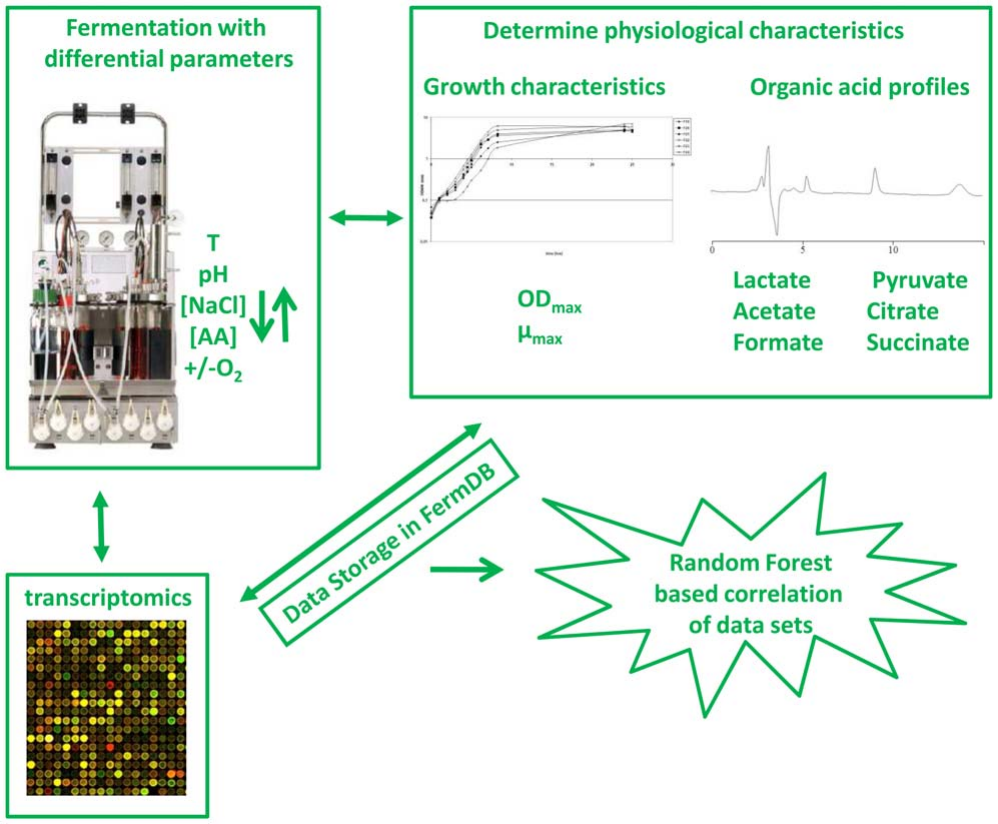
Workflow of the fermentation genomics platform. *L. plantarum* WCFS1 is fermented under different conditions and samples derived from these fermentations are assessed at the molecular level by full genome transcriptome profiling and in parallel the physiological characteristics of the fermentations are determined. The datasets obtained are stored in FermDB and correlated. Figure adapted from [8].

*L. plantarum* is encountered in dairy, vegetable, sourdough and meat fermentations [15], [22], [23]. Here, *L. plantarum* WCFS1 was grown under different conditions, according to a fractional factorial experimental design that included variations in temperature, NaCl concentration, pH, as well as oxygen and amino acid availability. Basic fermentation characteristics were monitored, such as maximal growth rate and biomass yield, as well as the respective transcriptome and organic acid profiles. This approach allowed the identification of fermentation conditions that modulate specific physiological characteristics of *L. plantarum* WCFS1, such as citrate uptake and succinate production, as well as the RF-based association of these characteristics with specific gene expression profiles. Moreover, our analysis revealed genetic markers that correlated with relatively high growth rates, and enabled regulatory network reconstruction that allowed the identification of overrepresented functional classes that explain the genetic response of *L. plantarum* to altered fermentation conditions such as pH or oxygen availability.

## Methods

### Experimental Design, Fermentations and Organic Acid Analysis

During the fermentations, *L. plantarum* WCFS1 [9] was grown in 2-fold concentrated chemically defined medium (2× CDM) [24] of which the composition can be found in Methods S1.To induce differential transcriptome and metabolite profiles in *L. plantarum* WCFS1 [9], a fermentation scheme was designed with 5 variable parameters, namely temperature (28 or 37°C), pH (5.2, 5.8 or 6.4), amino-acid concentration (1.1 or 2.0× standard amounts, see below), oxygen availability (sparging with N_2_ or air), and NaCl concentration (0 or 0.3 M). These variable fermentation conditions were combined into a combinatorial fermentation scheme on the basis of a balanced fractional factorial design (Table 1). Application of this experimental design reduced the number of fermentations to 24 (30 including controls, see below). Within this design, variations in fermentation conditions are distributed in such a way that the effects caused by the specific interaction of 2 different variable fermentation conditions can be quantified. As a trade off for the lower number of fermentations required, different combinations of three parameters are confounding and consequently their quantification is not feasible. pH-controlled batch fermentations were performed at 0.5 L scale in a Multifors mini-in parallel fermentor system (Infors-HT Benelux, Doetichem, the Netherlands). For inoculation of the fermentors, a single colony isolate of *L. plantarum* WCFS1 was used to inoculate 5 mL 2× CDM [24] and grown overnight at 37°C. This full-grown culture was used to prepare a dilution range from 10^−1^ to 10^−6^ in fresh 2× CDM medium, and these cultures were grown overnight. Subsequently, the cultures were photospectrometrically assessed and the culture with an OD_600_ nearest to 1.5 was used to inoculate the fermentors at an initial OD_600_ of 0.1. Prior to inoculation the 2× CDM media in the fermentors were adjusted to the appropriate pH and temperature. During fermentation the cultures were stirred at 125 rpm, the initial pH was maintained by the titration of 2.5 M NaOH, the OD_600_ was monitored continuously, and the cultures were sparged with N_2_ or air at a rate of 150 mL/min. Moreover, CO_2_ was mixed into these gasses at a final concentration of 2.5% via a mass flow controller prior to medium sparging, since this prevents the stagnation of growth of *L. plantarum* WCFS1 [25]. Throughout fermentations, OD_600_ was measured photospectrometrically, with the notion that when OD_600_ of undiluted samples exceeded 0.7, samples were diluted 10-fold in MRS medium prior to OD_600_ determination. After 25 h (stationary phase for all fermentations) 50 mL samples were collected for quantitative, high performance liquid chromatography (HPLC)-based organic acid analysis (pyruvate, acetate, citrate, succinate, formate and lactate levels were determined) according to a previously described protocol [26].

**Table 1:** Fermentation conditions and physiological characteristics of *L. plantarum* WCFS1 recorded for the 30 fermentors performed.

Ferm. ID:	[NaCl] (mM):	[AA]^f^:	T (°C):	pH:	O_2_/N_2_:	OD_max_:	µ_max_(h^−1^):
F1	0	2.0	37	5.2	N_2_	7.21	0.80
F2	300	2.0	37	5.2	N_2_	6.90	0.78
F3	0	1.1	37	5.2	O_2_	7.05	0.71
F4	300	1.1	37	5.2	O_2_	6.98	0.70
F5	0	2.0	28	5.2	O_2_	7.73	0.55
F6^a^	300	2.0	28	5.8	O_2_	6.03	0.55
F7	300	2.0	28	5.2	O_2_	7.35	0.44
F8^b^	0	1.1	28	5.2	N_2_	6.96	0.45
F9	300	1.1	28	5.2	N_2_	6.23	0.54
F10	0	2.0	37	6.4	O_2_	6.60	0.57
F11	300	2.0	37	6.4	O_2_	5.30	0.44
F12^c^	0	1.1	28	5.8	N_2_	6.60	0.48
F13	0	1.1	37	6.4	N_2_	6.56	0.58
F14	300	1.1	37	6.4	N_2_	6.07	0.53
F15	0	2.0	28	6.4	N_2_	5.85	0.31
F16	300	2.0	28	6.4	N_2_	4.16	0.23
F17	0	1.1	28	6.4	O_2_	7.74	0.32
F18^d^	0	2.0	37	5.8	N_2_	6.94	0.71
F19	300	1.1	28	6.4	O_2_	4.93	0.47
F20^d^	0	2.0	37	5.8	N_2_	5.01	0.76
F21	300	2.0	37	5.8	N_2_	4.68	0.75
F22	0	1.1	37	5.8	O_2_	5.92	0.65
F23	300	1.1	37	5.8	O_2_	6.07	0.60
F24^b^	0	1.1	28	5.2	N_2_	6.91	0.65
F25^e^	0	2.0	28	5.8	O_2_	6.14	0.29
F26^a^	300	2.0	28	5.8	O_2_	4.74	0.28
F27^c^	0	1.1	28	5.8	N_2_	6.23	0.24
F28	300	1.1	28	5.8	N_2_	4.84	0.23
F29^e^	0	2.0	28	5.8	O_2_	6.22	0.38
F30^d^	0	2.0	37	5.8	N_2_	5.94	0.71

Dotted lines separate fermentations performed on different days.

a–d duplicate fermentations on different days.

e duplicate fermentations on the same day.

f amino acids were added as 1.1 or 2.0× the standard amount in 2× CDM.

### RNA Isolation and DNA Microarrays

RNA isolation from *L. plantarum*, subsequent cDNA synthesis and indirect labeling, as well as DNA microarray hybridizations were performed using routine procedures [13], [27]. Briefly, 10 mL samples derived from the fermentors at an OD_600_ of 1.0 were quenched [28] prior to RNA isolation, and 5 µg of isolated RNA was used for cDNA synthesis and indirect labeling with cyanine 5 (Cy5) or cyanine 3 (Cy3) [13], [27]. The DNA microarray hybridization scheme was designed as a connected loop that consisted of smaller sub-loops containing all samples derived from fermentations that were run on the same day (Figure S1). A two-dye microarray-based gene expression analysis was performed on a custom-made 60-mer oligonucleotide array (Agilent Biotechnologies, submitted in the Gene Expression Omnibus (GEO) [29] under platform GPL13984) to determine genome-wide, absolute gene transcription levels. Co-hybridization of Cy5- and Cy3-labeled cDNA probes was performed on these oligonucleotide arrays at 42°C for 16 h in Slidehyb#1 (Ambion, Austin, USA). Subsequently, the slides were washed twice in 1× SSC containing 0.1% sodium dodecyl sulfate and twice in 1× SSC prior to scanning. Slides were scanned with a ScanArray Express 4000 scanner (Perkin Elmer, Wellesley, USA), and image analysis and data extraction were performed using the ImaGene Version 7.5 software (BioDiscovery Inc., Marina Del Rey, USA). The microarrays were scanned at different intensities and for each of the microarrays the best scan was selected on the basis of signal distribution (combination of a low number of saturated spots and a low number of low signal spots). The data were normalized using Lowess [30] normalization as available in MicroPrep [31]. The data were corrected for inter-slide differences on the basis of total signal intensity per slide using Postprep [31]. The median intensity of the different probes per gene was selected as the gene expression level. This analysis resulted in genome-wide, absolute gene expression levels for *L. plantarum* WCFS1 derived from 29 fermentations. CyberT [32] was used to compare and divide the transcriptomes in different possible classes (e.g. low vs high citrate, different levels in fermentation parameters), taking into account the duplicates (dye swaps) of each of the conditions. This analysis resulted in a gene expression ratio and false discovery rate (FDR) for each gene. Genes with FDR values <0.05 were considered to be statistically significant. All microarray data are MIAME compliant and available in the GEO database (GSE31076, http://www.ncbi.nlm.nih.gov/geo/query/acc.cgi?acc=GSE31076).

### Data Storage, Visualization Tools and Correlation Statistics

A MySQL-based storage system for data produced from the fermentation, transcriptomics and phenotyping experiments (e.g. the metabolite profiles presented here but also other functional characteristics such as gastrointestinal survival; see accompanying manuscript by van Bokhorst *et al*.) was developed. Moreover, the statistical methods Mann Whitney U [33] and Random Forest [16] were implemented for significance analysis and data correlation, respectively. More specifically, Mann-Whitney U [33] was used to correlate physiological characteristics of *L. plantarum* (µ_max_, OD_max_ and organic acid profiles) to differential fermentation conditions (temperature, pH, NaCl concentration, as well as oxygen and AA availability). Results were corrected for multiple comparisons using the false discovery rate control (FDR) [34]. To identify genes of which the transcription level correlated to (individual) citrate or succinate concentrations, the citrate and succinate results were divided into two different classes per compound (i.e. low citrate levels vs. high citrate levels, low succinate levels vs. high succinate levels) and subjected to RF using the transcriptome data as the predictor for the algorithm. From this prediction, the importance values (mean decrease gini) were used as a measure for functional correlation of the gene to the observed phenotype (Table S1). Using a similar RF-based approach, no clear target genes could be found on basis of transcript levels in relation to µ_max_ (data not shown). Therefore, a different approach was applied to identify µ_max_-related genes. In this alternative approach we grouped genes and calculated their average expression level per fermentation. Average gene expression of these grouped genes was compared to µ_max_ per fermentation by calculation of the Pearson correlation coefficient between the two. Per iteration the 300 groups that showed the best correlation with the µ_max_ of the fermentations were selected and the genes encompassing these groups were used for the next iteration step. Each subsequent iteration step differed from the previous with respect to group size (starting with a group size of 1, while group size increased by one gene in every consecutive iteration). As genes important for high correlation to µ_max_ will appear in multiple top-300 clusters, the total number of genes used for each next iterative step will decrease. This procedure was iterated until the total gene set did not further reduce in the subsequent iteration. The final gene set consisted of 47 genes. The expression levels of the genes in this final set was averaged per fermentation condition and correlated with the µ_max_. Both the storage system and the statistical methods have been integrated into a freely accessible, web-based platform designated FermDB (http://www.cmbi.ru.nl/fermdb). The platform is divided into two different Django applications termed “fermentations”, which includes all the storage and file handling procedures, and “analysis”, where all statistical analysis are handled.

DNA microarray data was visualized on the *L. plantarum*-specific metabolic model [35] using the Simpheny software package (www.genomatica.com). Functional overrepresentation was assessed using Biological Network Gene Ontology (BiNGO) [36] with the functional classification as proposed in [14] instead of GO annotation. To identify regulatory networks, the complete DNA microarray data set was grouped per individual fermentation parameter using CyberT. Genes with a significant change in expression (FDR <0.05) and a ratio change >1.5 fold were selected. A regulatory network was built that links variations in fermentation conditions to the significantly changed genes and visualized in Cytoscape. Within this network, genes were organized according to their association with specific fermentation conditions, as either single or shared responses towards certain fermentation conditions.

## Results

### Functional-fermentation Genomics Platform for *L. plantarum*

*Lactobacillus plantarum* WCFS1 was grown under a variety of conditions that differed in temperature, pH, as well as NaCl, amino acid, and O_2_ levels (Figure 1 and Table 1). The fermentations were performed on 5 separate days, whereas 1 and 4 control fermentations were included to investigate inter- and intra-day variability, respectively. Moreover, a triplicate fermentation for one of the intra-day controls (F18 and F20) was performed (F30) from which no samples were derived in the logarithmic phase of growth, confirming that sampling had no effect on growth parameters such as the maximum OD_600_ reached (OD_max_) and the maximum growth rate (µ_max_, Table 1).These three fermentations resulted in the highest OD_max_ and µ_max_ range observed in the complete fermentation scheme and appeared highly reproducible (OD_max_ = 5.94−6.94 and µ_max_ = 0.65−0.71). However, the other inter- and intra-day controls displayed distinct differences in OD_max_ and µ_max_, in particular when fermentation conditions were applied that induced suboptimal growth. Therefore, correlation analyses were performed on basis of the individual fermentations and their corresponding transcriptomes and organic acid profiles (see below).

The fermentation scheme resulted in an overall variation of the OD_max_ from 4.16 to 7.73 and a µ_max_ range from 0.23 to 0.80 h^−1^ (Table 1). The highest µ_max_ was achieved in fermentor F1 which was performed at pH 5.2 and 37°C, whereas the lowest µ_max_ was measured for fermentor F28 which was run at pH 5.8 and 28°C. The variations in these and other basic physiological characteristics such as lag phase (Figure S2A-E) strongly suggest that the fermentation scheme applied here will induce differential transcriptomes and corresponding phenotypes such as organic acid profiles, which were the variables measured for all samples derived from fermentors F1–F29. The transcriptome and phenotypic data were stored in fermDB which was specifically developed for this purpose (see Materials and Methods for details, accessible at http://www.cmbi.ru.nl/fermdb).

### Correlation of Physiological Characteristics to Specific Fermentation Conditions

Significant correlations were not found between AA availability and any of the physiological characteristics examined here (Table 2). Notably, glucose was not detected in any of the 29 fermentations after the cultures had reached the stationary phase of growth (data not shown), suggesting that in these carbon-limited fermentations the influence of the addition of surplus amounts of amino acids may only have a minor effect on the physiological characteristics of *L. plantarum* WCFS1.

**Table 2:** Mann-Whitney U-based correlation of fermentation parameters and physiological characteristics.

Parameter:	[lactate]:	[pyruvate]:	[acetate]:	[formate]:	[succinate]:	[citrate]:	OD_max_:	µ_max_ (h^−1^):
T	0.151	0.506	0.244	0.336	0.484	0.394	0.481	*0.022*
[AA]	0.321	0.337	0.331	0.491	0.505	0.432	0.242	0.260
O_2_	0.519	0.329	*0.022*	0.402	0.314	0.530	0.311	0.511
pH5.2 vs. 5.8	0.409	0.376	0.193	0.062	0.315	0.326	**0.014**	**0.016**
pH5.2 vs. 6.4	0.248	0.376	0.355	*0.016*	0.497	0.513	0.062	0.061
pH5.8 vs. 6.4	0.193	0.509	0.311	0.330	0.316	0.326	0.355	0.329
[NaCl]	0.326	0.506	0.245	0.505	**0.007**	*0.015*	**0.042**	0.349

Significant differences (p<0.05) are presented as italicized and bold, depending on the direction of the correlation (positive and negative, respectively).

**Figure 2:**
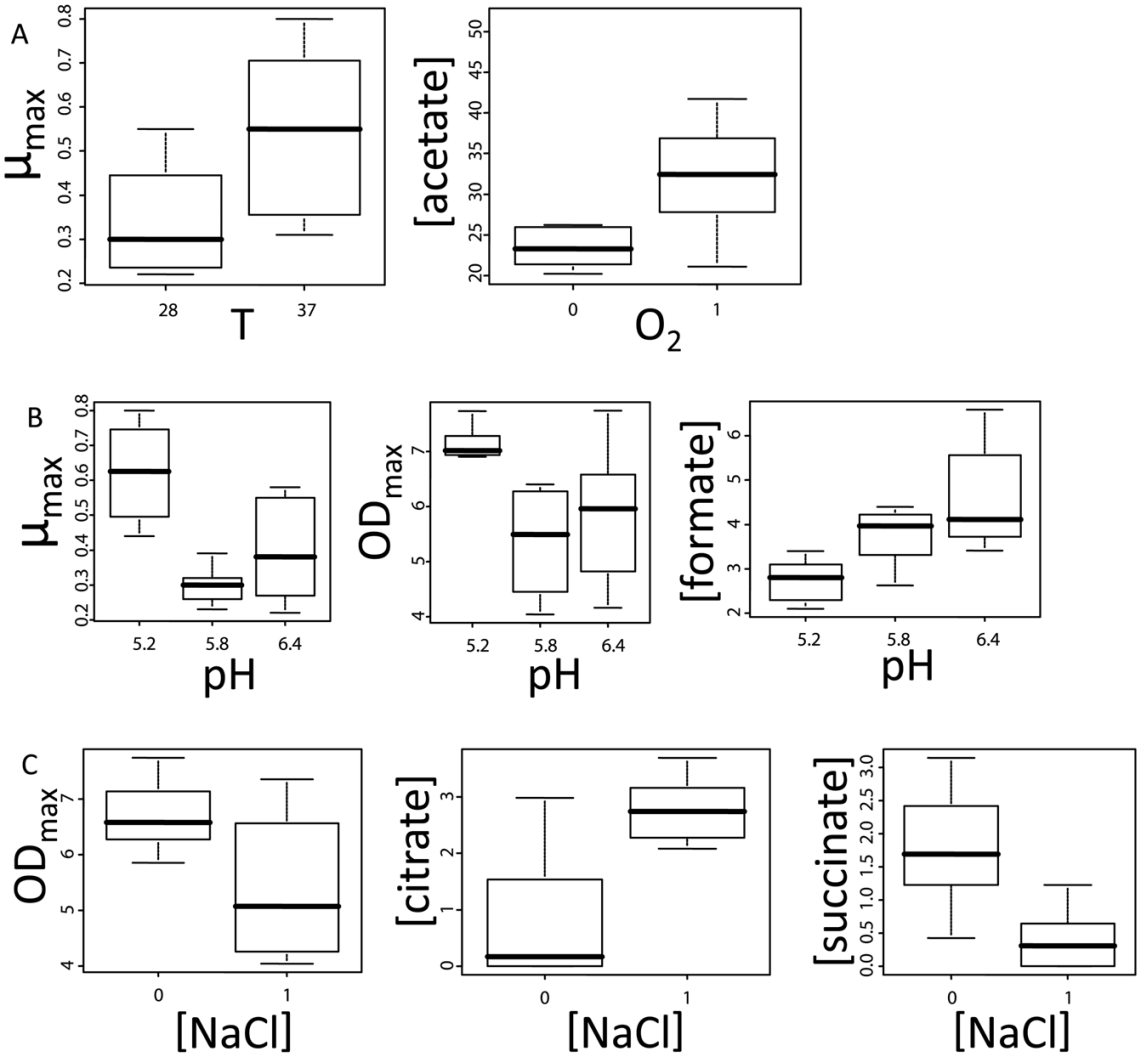
Box plots of the significant correlations in Table 2.

Not surprisingly, as it is well documented that *L. plantarum* grows well at 37°C [37], the average µ_max_ at this temperature was significantly higher than that recorded in the fermentations performed at 28°C (Table 2 and Figure 2A). Analogously, acetate concentrations were significantly higher in the aerobic fermentations. This might be explained by the fact that it is well established that only during aerobic growth lactate-to-acetate conversion can occur, with lactate remaining the major end product of carbon fermentation [38], [39], [40]. Notably, the lactate concentrations could not be associated with oxygen levels. This absence of a clear lactate effect might be due to the specific setup of this study. Considering the combinatorial nature of the fermentations performed, only fermentation variable-specific changes are anticipated to be identified. Lactate production, however, is probably influenced by many different fermentation variables and will therefore not be specifically linked to the availability of oxygen.

Interestingly, the NaCl concentration and pH in the fermentations could both be correlated with high significance to 3 physiological phenotypes of the cultures. When a pH of 5.2 was applied during fermentation, significantly lower amounts of formate were produced by *L. plantarum* when compared to fermentations at a pH of 6.4 (Table 2). A similar trend was found comparing fermentations at pH 5.2 and 5.8 (p = 0.062), suggesting an overall correlation between pH and formate concentrations (Figure 2B). Secondly, fermentation at pH 5.2 resulted in significantly higher µ_max_ and OD_max_ relative to the fermentations which were maintained at the other two pH values. The OD_max_ reached by *L. plantarum* WCFS1 was consistently and significantly lower in fermentations to which NaCl was added (Table 2 and Figure 2C). Thirdly, NaCl addition also yielded decreased and increased concentrations of succinate and citrate, respectively. Overall, our data corroborate and expand earlier described correlations between physiological characteristics and fermentation conditions, despite the fact that multiple variables were changed concomitantly, confirming the feasibility of our approach.

### Transcriptome Correlation to Physiological Parameters

**Figure 3:**
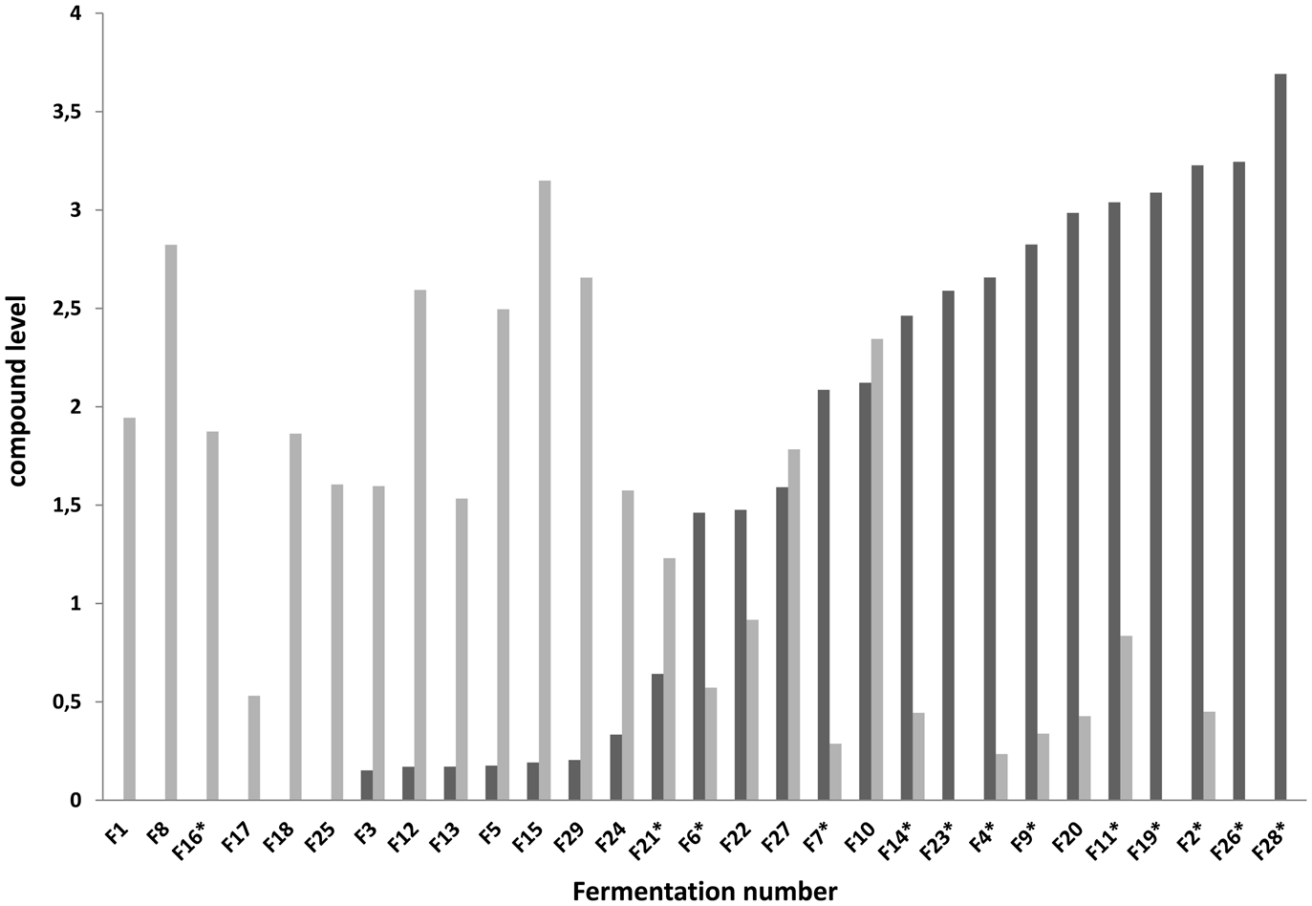
Citrate (dark bars) and succinate (light bars) concentrations in the 29 fermentations. Fermentors marked with an asterisk represent fermentations to which 0.3 M NaCl was added.

The citrate and succinate concentrations determined in the 29 fermentations appeared to anti-correlate (R^2^ = −0.77). Comparison of the citrate concentrations at the start and after 25 h of incubation indicate that citrate is taken up by the cells and subsequently converted to succinate, particularly in fermentations with low NaCl concentrations (Figure 3). A RF-based identification of transcripts that are associated with citrate or succinate concentration revealed that among the 10 most important genes for the classification of citrate and succinate, 5 genes were found shared between the two predictions (*lp_0603*, *lp_1112*, *lp_1562*, *lp_2451* and *lp_2825*). In addition to the RF classification, the same class distribution (low vs high) was used to assess the gene expression (Table S1). Out of the 3099 genes represented on the DNA microarray, 415 showed to have a significant up/down regulation (p<0.05) in both class distributions. Out of these 415 genes, 407 showed to be expressed in the same direction (e.g. upregulated at high citrate uptake and high succinate production), again confirming the highly inter-connectiveness of these two metabolites. Among these significantly changed genes were all 5 shared top-10 genes resulting from the RF, each showing a ratio of expression above 1, indicating a higher expression of these genes in the case of citrate to succinate conversion. Among these genes only one was directly related to the conversion of citrate to succinate (*lp_1112*, encoding a fumarate hydratase). For the other genes this relation is less clear (e.g. *lp_0603*, coding for a putative acetyltransferase). Interestingly, among these five genes two appear to be involved in nucleotide biosynthesis, more specifically in the conversion of (preferably) uridine.

**Figure 4:**
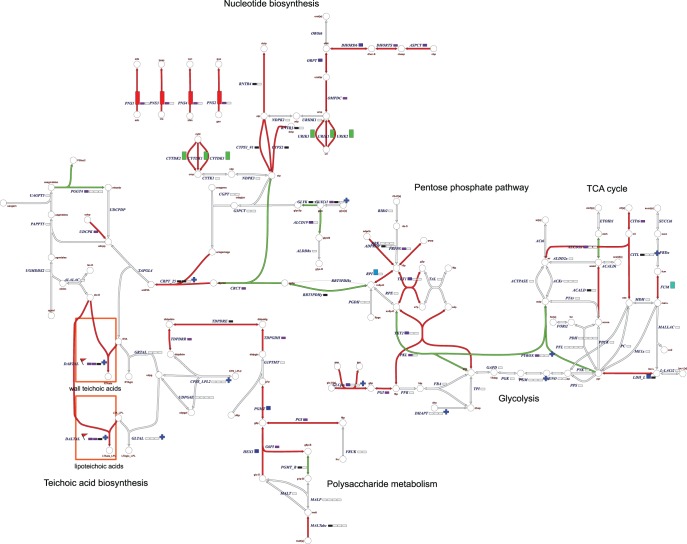
Simpheny-based visualization of the metabolic map of *L. plantarum* WCFS1 reveals the interconnectivity of the pentose phosphate pathway, and teichoic acid and nucleotide biosynthesis to citrate and succinate levels. Arrows indicate reaction and their up- or downregulation (p<0.05 in green and red, respectively) in the increased citrate uptake and succinate production conditions. Marked with boxes next to the reaction names is the average importance per gene involved in the reaction for the two classifications (citrate and succinate). Bar heights correspond to importance levels.

The transcriptome data and the RF importances were averaged for the two classifications (taking the average gene expression ratio/importance from both the citrate and succinate classes) and plotted on the genome scale metabolic map in Simpheny (see methods and Table S1). Apart from the genes described earlier, we identified additional genes in the metabolic vicinity of citrate to succinate conversion and nucleotide biosynthesis to be altered in expression and/or important for RF classification (Figure 4). In addition to these regions in the metabolic map, changes were observed in teichoic acid biosynthesis.

**Figure 5:**
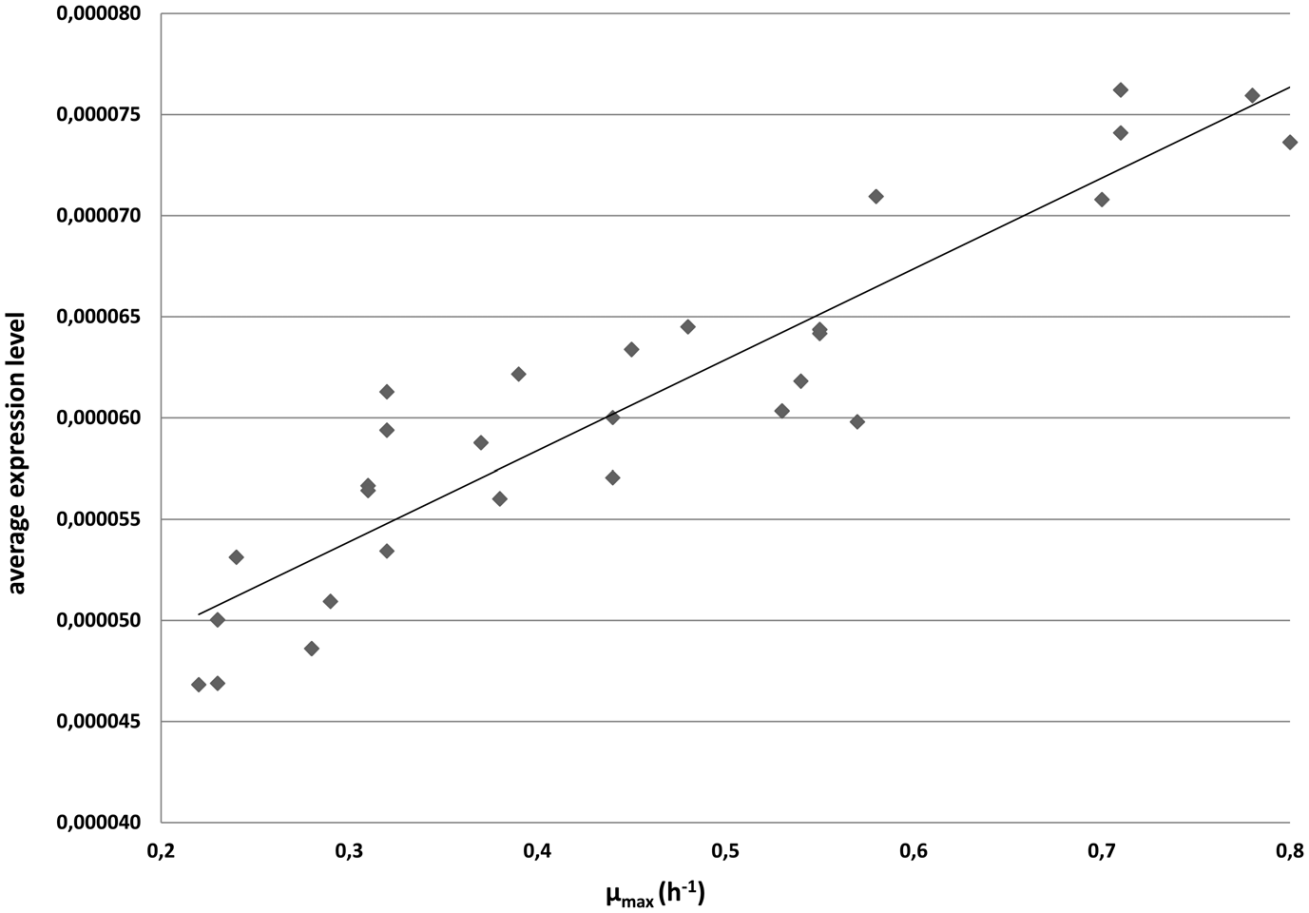
Scatterplot of the µ_max_ and expression level of the highly correlated genes. Each diamond symbol in the plot corresponds to a single fermentation or which both µ_max_ and microarray data was determined. Expression levels were normalized (see methods) and averaged. Correlation of µ_max_ and transcriptome data is 0.95 (Spearman rank).

Using a similar RF-based approach, no clear target genes could be found on basis of transcript levels in relation to µ_max_ (data not shown), most probably due to the relative low signal to noise ratio within the transcriptome data. An alternative approach using groups of genes rather than single genes (see Methods) resulted in a highly significant correlation (R^2^ = 0.95) of the average expression level of 47 genes and µ_max_ (Figure 5 and Table S2). The encoded functions of these genes include 8 regulators from diverse families, 13 hypothetical and 4 transport-related proteins, 8 functions involved in energy and central intermediary metabolism, and 5 genes that play a role in cell envelope functionalities.

Overall, these analyses demonstrate the suitability of our genomics fermentation platform to identify genetic biomarkers for highly (industrially) relevant physiological characteristics of the corresponding cultures, such as growth rate and organic acid profiles.

### Identification of Regulatory Networks

**Figure 6:**
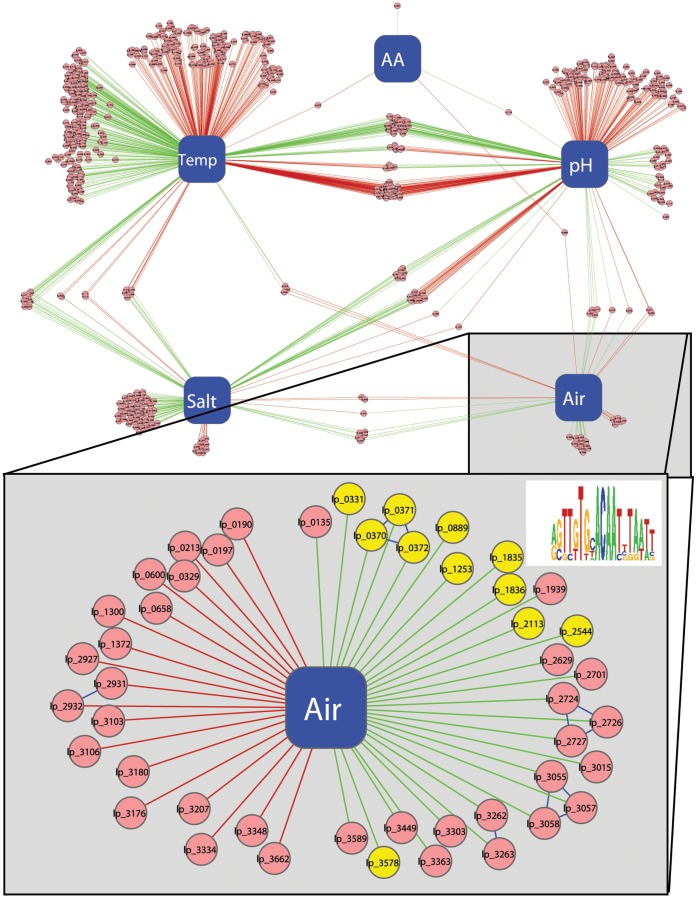
Reconstruction of the transcriptome response clouds to (combinations of) different fermentation parameters. Nodes in the network correspond to the fermentation parameters (in blue) and the genes with significant (p<0.05) altered (ratio change >2) expression level (light red). Each gene for which gene expression is significantly altered by changing the fermentation parameter is connected to that fermentation parameter by an edge. Edge colour reflects under which condition the gene has the (relatively) highest expression (red = low temperature, [NaCl], [AA], pH or O_2_ levels or green = high temperature, [NaCl], [AA], pH or O_2_ levels). Visualization of the network was performed in cytoscape (http://cytoscape.org). Genes are grouped on the basis of shared response with the different fermentation variables. In the lower panel the response cloud of O_2_ is shown in more detail, in combination with a web logo of the putative TF binding site. Genes that share this binding site in their upstream sequence have been colored yellow.

Construction of regulatory networks revealed that, in line with the fact that amino acid concentrations had no impact on the physiological characteristics of *L. plantarum* (see above), virtually no transcriptome responses were observed for this fermentation-variable (Figure 6). For the other 4 fermentation-variables, significant transcriptome responses (response clouds) were observed in both directions (e.g. up- and down-regulation of genes). The largest response clouds were observed for temperature and pH, including a significant proportion of shared responses.

To assess which functional classes are overrepresented in the observed response clouds, a BiNGO analysis was performed (Table S3). For the low-temperature response cloud only a single functional class was found to be overrepresented (p = 0.042), encompassing the regulatory functions. The functional class found as most significantly overrepresented (p = 5.6×10^−35^) in the high-temperature response cloud encompasses phage- and prophage-related functions, whereas TCA cycle and fermentation were also overrepresented significantly. Despite the fact that the above mentioned functional classes are significantly overrepresented, it is hard to assess their role in the determination of the functional properties and adaptation of *L. plantarum* to the altered fermentation conditions.

The functional classes found overrepresented in the salt-response clouds include several sub-classes associated with amino acid and nucleotide biosynthesis and transport (Table S3), reiterating our earlier observations that the addition of salt to the fermentation media correlates with increased citrate uptake and succinate production, which according to our Simpheny analyses involved several amino acid- and nucleotide-related processes (see above).

Interestingly, among the two individual oxygen response clouds (up- and down-regulated) nucleotide biosynthesis was identified as an overrepresented class. Detailed analyses of these two response clouds pinpointed that, although the class was shared, the clouds consisted of different genes. Manual inspection of these genes revealed that under conditions that lacked oxygen, *lp_2931* and *lp_2932* were consistently upregulated. These genes code for NrdD and NrdG, which are class III ribonucleotide reductases, a class of ribonucleotide reductases that only function under anaerobic conditions [41], [42]. Furthermore, it was shown in different organisms, including LAB, that these genes are essential for growth under anaerobic conditions [42], [43]. Reduction of these ribonucleotides under anaerobic conditions could function as a source for deoxyribonucleoside triphosphates for DNA sysnthesis. This could also explain why under aerobic conditions, parts of the nucleotide biosynthesis pathways (i.e. *purL* (*lp_2724*), *purS* (*lp_2726*), *purC* (*lp_2727*) and *pyrAA* (*lp_2701*)) were found to be induced.

In addition to the nucleotide biosynthesis, we also found other genes that were clearly either negatively or positively correlated to differences in the availability of oxygen during fermentation. Among these genes, we identified several typical functions involved in oxygen metabolism in *L. plantarum* that appeared to be consistently induced in presence of oxygen, e.g., pyruvate oxidases (*pox3* (*lp_2629*), *pox5* (*lp_3589*)) and NADH oxidases (*nox5* (*lp_3449*)) as well as members of the oxygen-stress regulon like catalase (*kat* (*lp_3589*), NADH peroxidase (*npr2* (*lp_2544*)), protein methionine S-oxide reductase (*msrA2* and *mrsB* (*lp_1835* and *lp_1836*)) and a glutathione reductase (*gshR2* (*lp_1253*). Conversely, several genes involved in fermentation appeared to be expressed at a lower level in the presence of oxygen, like alcohol dehydrogenase (*adhE; lp_3662*) and acetaldehyde dehydrogenase (*acdH*; *lp_0329*), which may very well relate to their role in maintenance of the redox balance during anaerobic fermentation [44].

Detailed analysis of the upstream regions of the genes in the O_2_ response cloud revealed the presence of a putative transcription factor (TF) binding site with a clear inverted repeat consensus sequence (5′-agTTGTGCACAAtttaatt-3′) upstream of a large number of genes elevated in expression under the O_2_-rich condition (Figure 6). This TF binding site was previously identified in a study that compared a large number of different microarray experiments in *L. plantarum* [45], but was not linked to presence of O_2_ under these conditions, likely caused by the fact that the dataset used lacked sufficient description of the experimental setup. These results clearly establish that the approach chosen enables the generic modulation of multiple genes and pathways by the variation of fermentation conditions, and that genomic methodologies enable their molecular recoding as well as the comprehensive mining of their inter-relations.

A relative large number of genes in the response cloud identified for lower pH levels represent the main functional class “transport and binding” (10 out of 29 genes, p = 0.04). Among these genes, many different subclasses of transport and binding were found (amino acid, organic acids, cations, anions and unknown substrates) and did not represent any single cellular pathways with clear relation among the proteins. However, the response clouds to the combination of pH with other fermentation parameters; salt (downregulated), and temperature (down- and up-regulated) all show a clear overrepresentation of different functional subclasses of the “transport and binding” class (cations, anions, and amino acids, respectively). These findings suggest that multiple pH-adaptation mechanisms exist in *L. plantarum* WCFS1, and that the executed pH adaptation depends on other fermentation conditions besides the pH, whereas membrane potential alterations appear to correlate to metabolite transport.

In conclusion, these regulatory networks provide insight into the genetic factors underlying correlations between (combinations of) fermentation conditions and physiological characteristics of *L. plantarum.*

## Discussion

This study reports an integrated approach in which *L. plantarum* WCFS1 was fermented under different conditions, followed by assessment of the physiological characteristics of the cells and full-genome transcriptomes (Figure 1). All data obtained were stored in a correlation database that is freely accessible, in a web-based platform designated FermDB. The fermentation scheme used here was based on a balanced fractional, factorial design whereby multiple parameters were varied in between fermentations. This design reduced the number of fermentations required (24 excluding controls) compared to 1 by 1 variation of these parameters (32 fermentations excluding controls). Yet, even with this reduced scheme, the effect of varying combinations of two parameters in the fermentations could still be clearly established. Moreover, the fact that single fermentative parameters could be readily associated with physiological parameters in the background of multiple variables between fermentations enhances the likelihood that the data generated here can be extrapolated to other fermentation media and/or conditions, including industrially relevant fermentation settings. Correlation analysis of the data pinpointed the fermentation conditions that are of importance for the discrimination of particular physiological characteristics, such as maximum growth rate, biomass yield, and organic acid profiles. Furthermore, the expression levels of specific genes could be associated with physiological characteristics, providing insight into the molecular mechanisms involved.

Our approach readily revealed that physiological characteristics of *L. plantarum* are dramatically influenced by the fermentation conditions. For example, the capacity to convert citrate to succinate depended on the salt concentration applied during fermentation and had a clear effect on the maximum OD_600_ reached in the fermentation. Citrate to succinate conversion by *L. plantarum* and several other LAB has been documented previously and was demonstrated to be coupled to the degradation of lactic acid [46], [47]. In this process, lactate is degraded to acetate via a pyruvate formate lyase, where oxaloacetate (formed from citrate) is used as an electron acceptor, resulting in the production of succinate, formic acid and CO_2_. Degradation of lactate into acetate yields an additional ATP from the original carbon source [26], [46] and could therefore explain the increased yield observed in the fermentations without added salt. Unfortunately, we did not observe an increase in acetate and formic acid production and lactate utilization, probably due to the interference of other fermentation variables (formic acid production was significantly different between fermentations at different pH levels, while acetate production was significantly different between aerobic vs anaerobic conditions). Although citrate to succinate conversion has been previously studied in *Lactobacillus plantarum*, no fermentation conditions were identified that influence the utilization of citrate. In this study we show that the addition of NaCl to the fermentation significantly affects citrate to succinate conversion independent of other fermentation variables. As this change in citrate to succinate conversion was coupled to a decreased maximum OD_600_, it is likely that the complete lactate degradation cascade is not functional anymore when NaCl is added to the medium. Most likely, this decrease in citrate utilization is coupled to changes in the capacity of the cells to efficiently transport citrate across the membrane, due to the changes in osmolarity caused by the addition of NaCl to the medium. Notably, the concomitant transcriptome profiles obtained also pinpointed to the effect of salt on cell envelop associated functions, including teichoic acid biosynthesis.

Besides the correlation of physiological characteristics and fermentation discussed above, our fermentation genomics platform enables the association of fermentation conditions and transcriptome profiles, e.g. the O_2_ response cloud as presented in Figure 6. Detailed analyses of the genes in the O_2_ response cloud showed the presence of only a single gene annotated as a transcriptional regulator (*lp_0889*). This regulator is annotated as a member of the MarR family, a family of proteins involved in the regulation of virulence factor production, catabolism of environmental aromatic compounds and response to antibiotic and oxidative stresses [48]. In *Bacillus subtilis* OhrR, a member of the MarR family, was shown to be involved in the regulation of the expression of *ohrA*, a gene involved in the protection against different reactive oxygen species [49]. In this study, the binding site for OhrR was determined as an inverted repeat with the sequence TACAATT-AATTGTA. Comparison of the *B. subtilis* inverted repeat with the predicted binding site of the *L. plantarum* genes in the O_2_ response cloud showed a high resemblance of these two sequences; the first part of the inverted repeat upstream of genes in the O_2_ response cloud resembles the second part of the OhrR bindingsite (TTGTG versus AATTGTA) and vice versa (CACAA versus TACAATT). Considering these observations, we hypothesize that Lp_0889 is a regulator of O_2_ response in *Lactobacillus plantarum* WCFS1.

The majority of the transcript-phenotype correlations identified involve genes that are conserved within the species *L. plantarum*, which prevents them from being identified by gene-trait matching approaches [14]. This observation exemplifies the complementarity of the transcriptome-trait matching approach presented here. Moreover, genetic biomarkers identified by gene-trait matching are not necessarily present in industrially applied strains [15], which hampers the gene-based improvement of strain performance. Industrial strains are generally selected on basis of a combination of traits, e.g. acidification rate [50], phage resistance [51], flavor-formation [2], [3], [6], [7] or probiotic functionality [1], [52], in combination with robustness under industrial processing conditions, e.g. freeze- or spray-drying procedures [8], [53]. Therefore, our transcriptome-trait matching results seem to have a broader applicability as compared to gene-trait matching approaches, since transcriptome-trait matching allows fermentation-enhanced improvement of a specific trait whilst applying the same strain. Conversely, the industrial implementation of gene-trait matching results might require tedious selection of alternative strains on basis of identified functional markers whilst attempting to maintain other functionalities.

This paper exemplifies the feasibility of FermDB to correlate transcriptome profiles to physiological characteristics or fermentative parameters of *L. plantarum*. However, this bioinformatic suite is currently implemented for broader exploitation. For instance, the availability of transcriptomics data under a plethora of conditions can facilitate the selection of engineered gene deletion derivatives by applying growth conditions that do not require the targeted gene to be expressed. Moreover, this strategy has recently led to the successful reversion of phenotypes initially observed in *L. plantarum* cell-envelope mutants by modification of the fermentation medium utilized [54]. FermDB has also been expanded with new functional *L. plantarum* WCFS1 datasets such as gastrointestinal survival (see accompanying manuscript by van Bokhorst *et al*.), resulting in the identification of fermentation conditions and genetic markers for these industrially relevant characteristics as well. Notably, the fermentation genomics strategy can be readily applied for other LAB species. Ultimately, these approaches enable the linkage between genes and functions which will further improve genome annotations. In parallel, it will provide a knowledge platform that can enable the rational design of industrial fermentation and process conditions for the production of LAB with improved functional characteristics.

## Supporting Information

Figure S1 **DNA microarray hybridization scheme**. F1–F29 represent the fermentations as presented in Table 1. Tail and head of the arrow represent Cy3 and Cy5 labeling, respectively. All subloops are labeled as the F1–F6 subloop.
(PPT)

Figure S2 **S2A-E display the growth curves of the 30 fermentations performed on 5 separate days.**
(PPTX)

Methods S1 **Detailed description of the medium composition of 2× CDM.**
(DOC)

Table S1 **RF importances and transcript levels per gene for succinate production and citrate consumption levels.** Bold genes represent shared top-10 importance factors for citrate and succinate.
(XLSX)

Table S2 **47 genes of which the average transcription level correlates with µ_max_.**
(XLSX)

Table S3 **BiNGO analyses to identify the functional gene categories overrepresented in response to modifications in fermentative parameters.**
(XLSX)
