# Supplementary figures and images for: Transcriptomes Reveal Genetic Signatures Underlying Physiological Variations Imposed by Different Fermentation Conditions in Lactobacillus plantarum
Source: PLoS One. 2012 Jul 3;7(7):e38720. doi: 10.1371/journal.pone.0038720 (PMC3389018; doi:10.1371/journal.pone.0038720)

## Slide 1
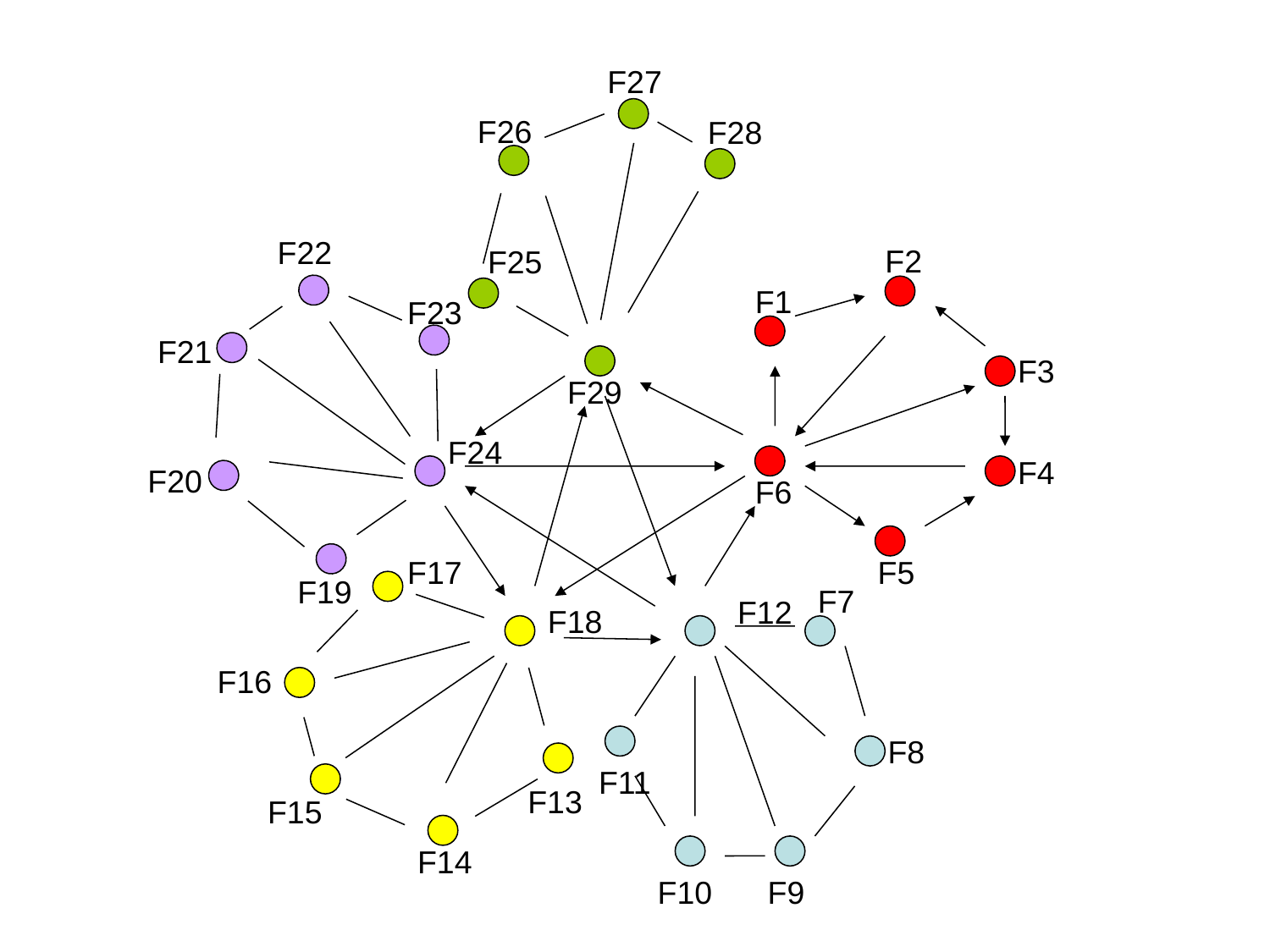

F27
F26
F28
F22
F2
F25
F1
F23
F21
F3
F29
F24
F4
F20
F6
F17
F5
F19
F7
F12
F18
F16
F8
F11
F13
F15
F14
F10
F9

Supplement: Figure S1 — DNA microarray hybridization scheme. F1–F29 represent the fermentations as presented in Table 1. Tail and head of the arrow represent Cy3 and Cy5 labeling, respectively. All subloops are labeled as the F1–F6 subloop. (PPT) [file pone.0038720.s001.ppt]
