# Supplementary material for: Transcriptomes Reveal Genetic Signatures Underlying Physiological Variations Imposed by Different Fermentation Conditions in Lactobacillus plantarum
Source: PLoS One. 2012 Jul 3;7(7):e38720. doi: 10.1371/journal.pone.0038720 (PMC3389018; doi:10.1371/journal.pone.0038720)
Supplement: Figure S2 — S2A-E display the growth curves of the 30 fermentations performed on 5 separate days. (PPTX) [file pone.0038720.s002.pptx]

## Slide 1
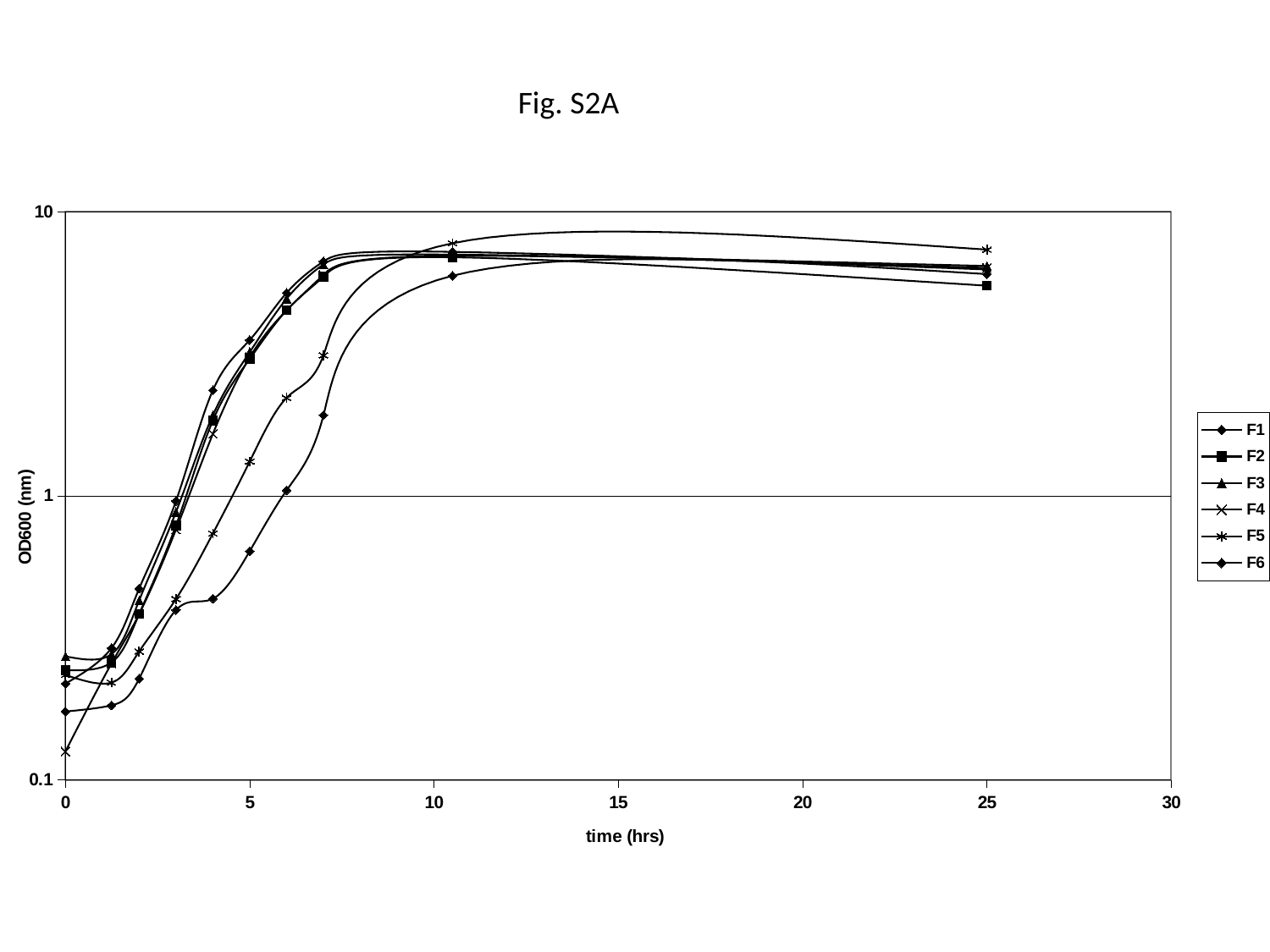

Fig. S2A
### Chart
| Category | F1 | F2 | F3 | F4 | F5 | F6 |
|---|---|---|---|---|---|---|

## Slide 2
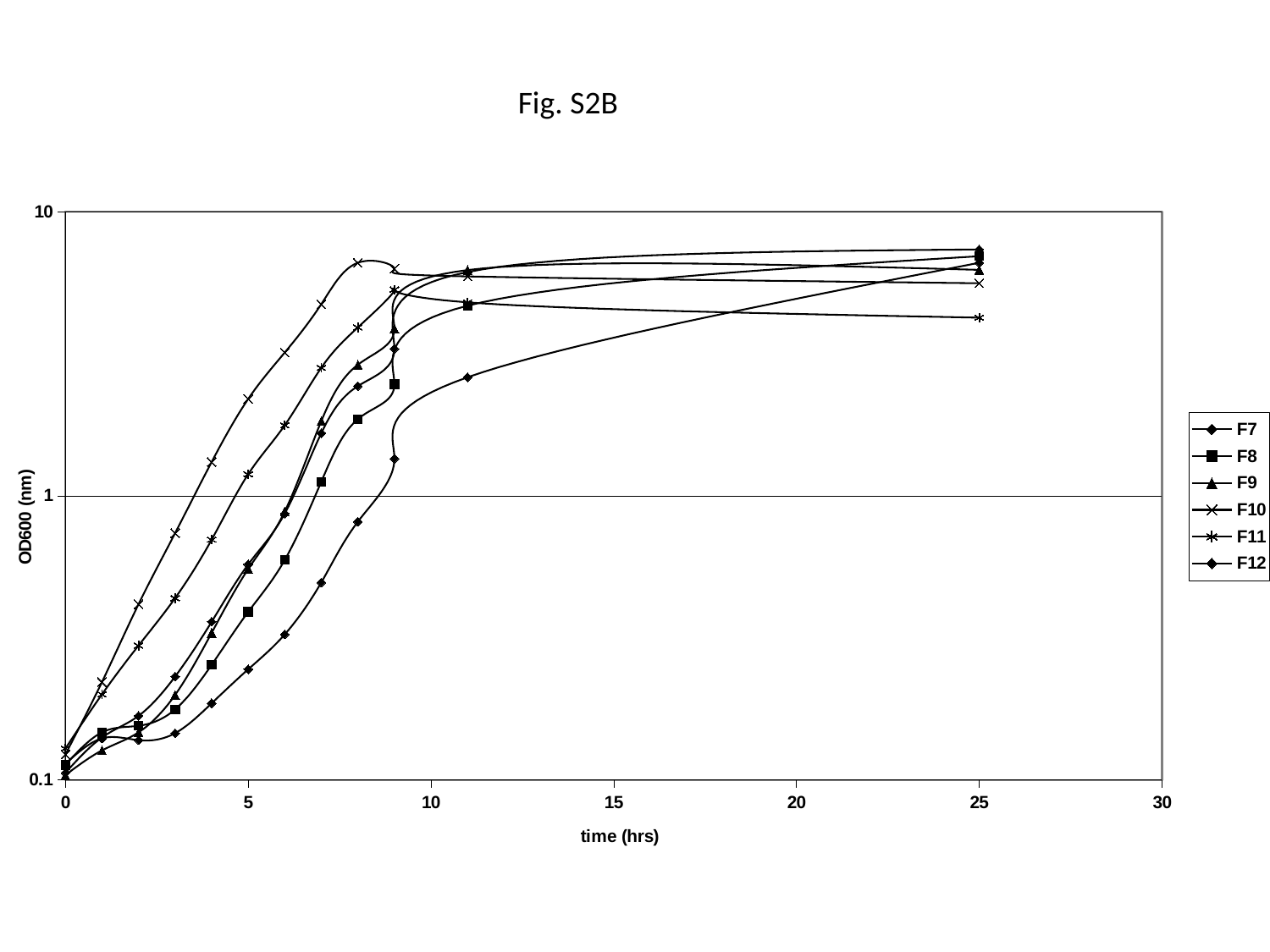

Fig. S2B
### Chart
| Category | F7 | F8 | F9 | F10 | F11 | F12 |
|---|---|---|---|---|---|---|

## Slide 3
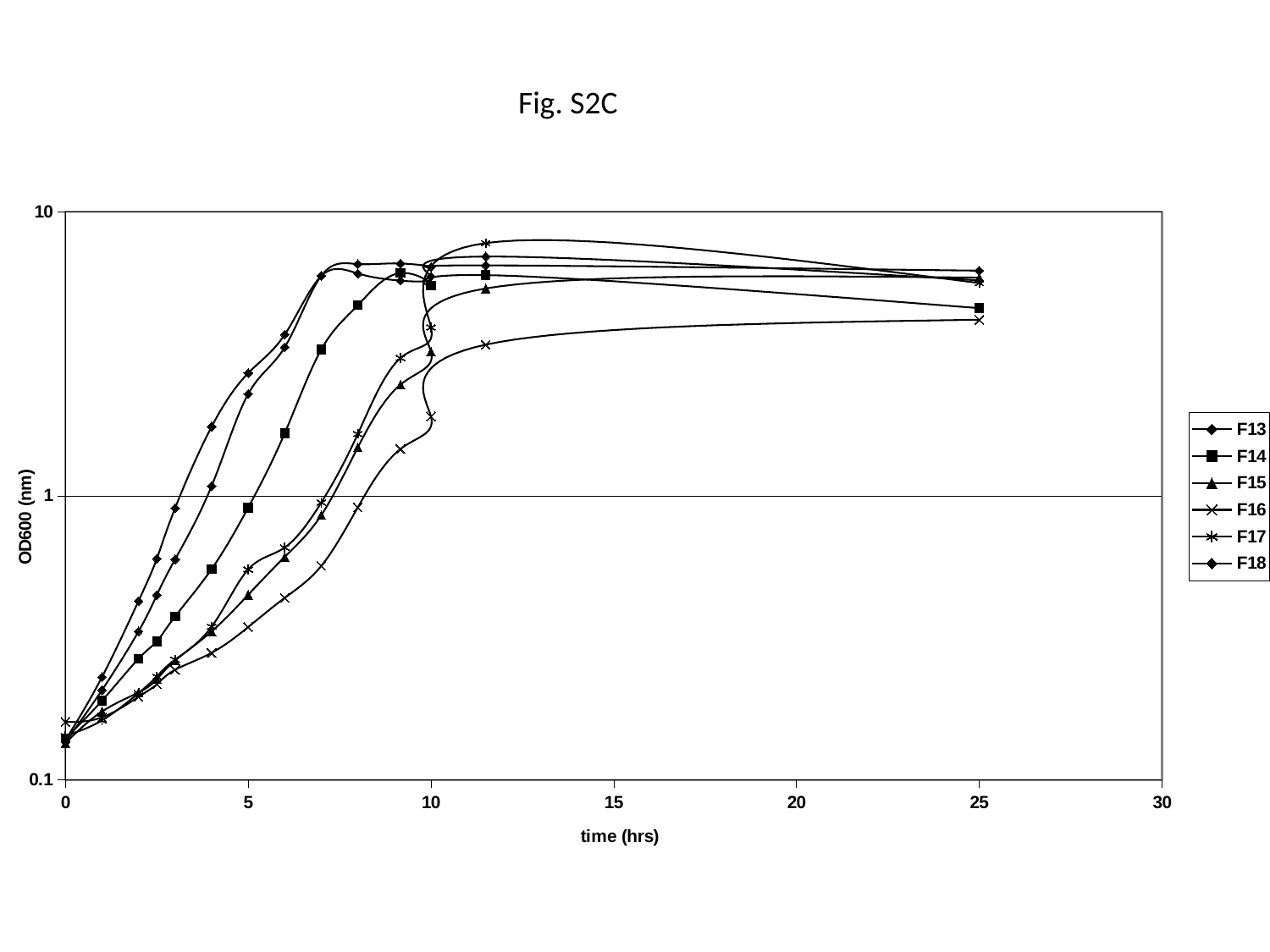

Fig. S2C
### Chart
| Category | F13 | F14 | F15 | F16 | F17 | F18 |
|---|---|---|---|---|---|---|

## Slide 4
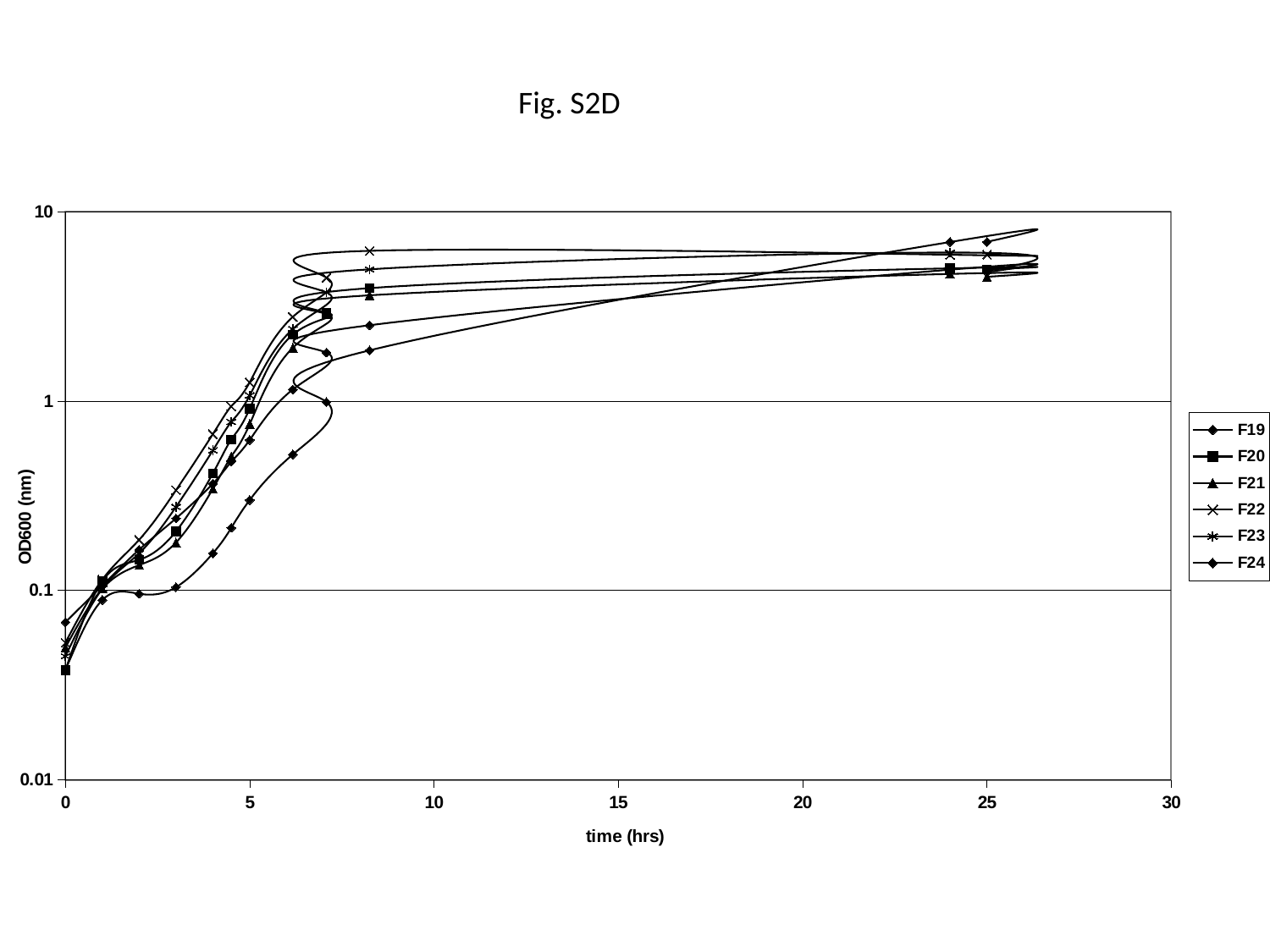

Fig. S2D
### Chart
| Category | F19 | F20 | F21 | F22 | F23 | F24 |
|---|---|---|---|---|---|---|

## Slide 5
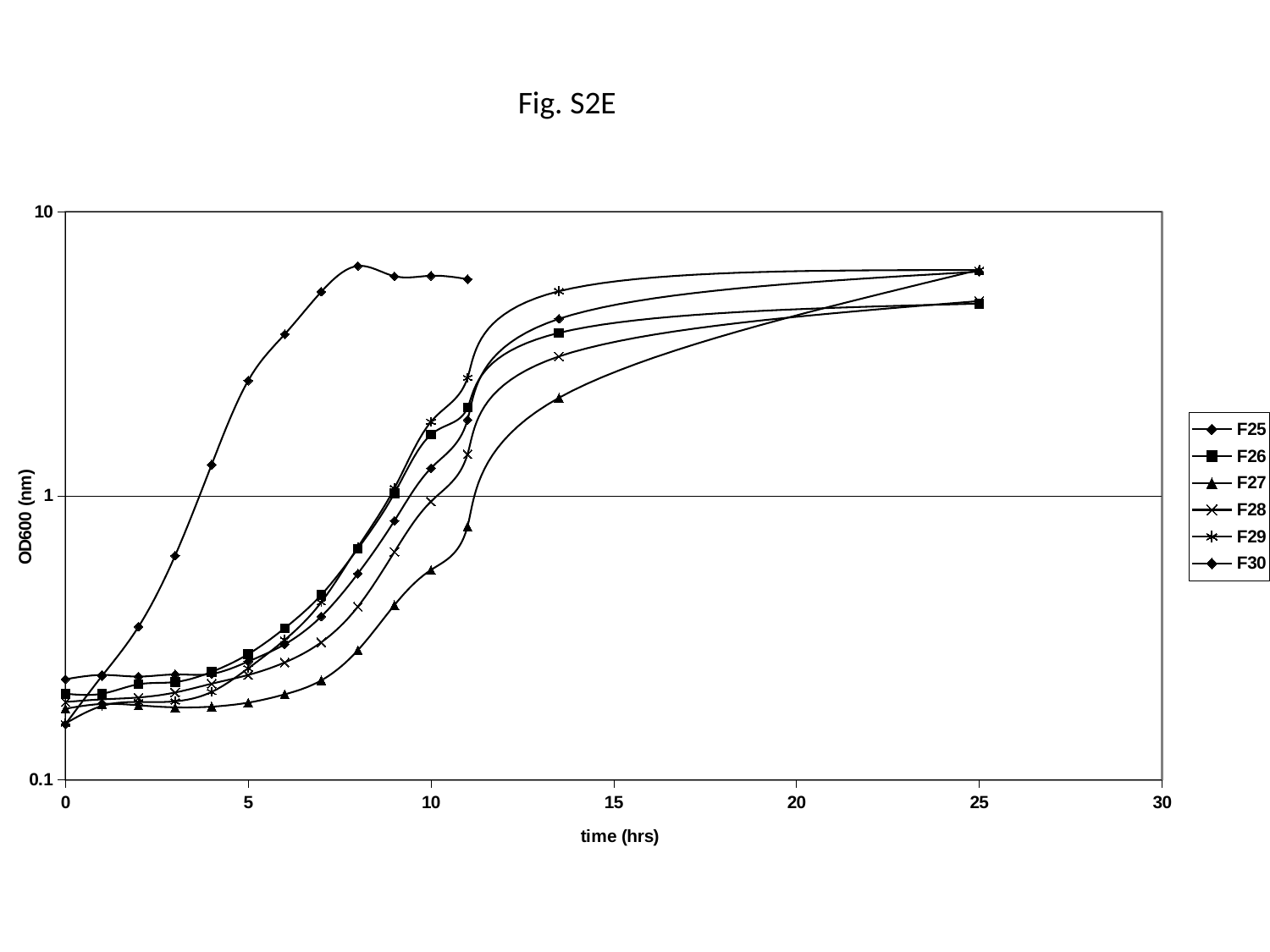

Fig. S2E
### Chart
| Category | F25 | F26 | F27 | F28 | F29 | F30 |
|---|---|---|---|---|---|---|
