# Supplementary material for: Transcriptomes Reveal Genetic Signatures Underlying Physiological Variations Imposed by Different Fermentation Conditions in Lactobacillus plantarum
Source: PLoS One. 2012 Jul 3;7(7):e38720. doi: 10.1371/journal.pone.0038720 (PMC3389018; doi:10.1371/journal.pone.0038720)
Supplement: Methods S1 — Detailed description of the medium composition of 2× CDM. (DOC) [file pone.0038720.s003.doc]

**Supplementary Methods**

**1L of 2x *L. plantarum* chemically defined medium (2x CDM) for fermentations based on Cremoris Defined Medium (see references below)**

Dissolve in 650 ml distilled water: Supplier + product number:

- 2 g K_2_HPO_4_ Merck 4873

- 10 g KH_2_PO_4_ Merck 5104

- 3.32 g Na-acetate.3H_2_O Merck 6268

- 1.2 g (NH_4_)_3_-citrate BDH 27153

- 0.5 g tyrosine (dissolves poorly) Sigma T-3754

- 15 g glucose-monohydrate Scharlau GL0129100

- 1 g L(+)Ascorbic acid (vit. C) Sigma A-0278

Add mix solutions (see below for composition):

- 200 ml 10x ami­no acid solution

- 20 ml 100x DNA precursor mix

- 20 ml 100x metal solution

- 20 ml 100x vitamin solution

Adjust volume to 1L

Adjust pH to desired value

Filter sterilise solution over a 0.22 µm filter

# MIX solutions

**100x DNA precursor mix**

10 mg adenine Sigma A-8626

10 mg guanine Sigma G-0381

10 mg xantine Sigma X-7375

10 mg uracil Sigma U-114

dissolve in 10 ml 0.1 M NaOH

Store in –40°C freezer (stable for months)

**100x Vitamin solution**

Per litre distilled water

- 500 mg pyridoxamine-HCl Sigma P-9380

- 250 mg D-biotin Sigma B-4501

- 250 mg 6,8-thioctic acid Sigma T-5625

- 200 mg pyridoxine-HCl Sigma P-9755

- 100 mg nicotinic acid Sigma N-0765

- 100 mg Ca-(D+)pantothenate Sigma P-6045

- 100 mg Riboflavin Sigma R-4500

- 100 mg thiamin-HCl Sigma T-4625

- 100 mg vitamin B12 Sigma V-2876

- 100 mg folate Sigma F-7876

- 1 g p-aminobenzoëic acid Sigma A-0129

- 500 mg orotic acid Sigma O-2625

- 500 mg thymidine Sigma T-5018

- 500 mg inosine Sigma I-4125

Adjust pH to 10 by addition of 10 M NaOH to dissolve all the vitamins

Adjust pH to 6.8

Store in –40°C freezer (stable for months)

**100x Metal solution**

Per litre distilled water:

- 20 g MgCl_2_  6 H_2_O BDH 10149

- 5 g CaCl_2_  2 H_2_O Merck 2382

- 1.6 g MnCl_2_  4 H_2_O BDH 10152

- 0.3 g FeCl_3_  6 H_2_O Sigma F-2877

- 0.5 g FeCl_2_  4 H_2_O Sigma F-2130

- 0.5 g ZnSO_4_  7 H_2_O BDH 10299

- 0.25 g CoSO_4_  7 H_2_O Merck 2546

- 0.25 g CuSO_4_  5 H_2_O BDH 10091

- 0.25 g (NH_4_)_6_Mo_7_O_24_  4 H_2_O BDH 10028

Dissolve the FeCl_2_  4 H_2_O in 10 ml 17 % HCl and FeCl_3_  6 H_2_O in 200 ml water, dissolve all other components in 700 ml water. After all three solutions are dissolved add them together and make the final volume 1000 ml.

Store in –40°C freezer (stable for months)

**10*Amino acid solution**

Per litre distilled water:

- 2.40 g alanine Sigma A-7627

- 1.25 g arginine Sigma A-5006

- 4.20 g aspartic acid Sigma A-9256

- 1.30 g cysteïne-HCl Fluka 30120

- 5.00 g glutamic acid Sigma G-1251

- 1.50 g histidine Sigma H-8000

- 2.10 g isoleucine Sigma I-2752

- 4.75 g leucine Sigma L-8000

- 4.40 g lysine Sigma L-5626

- 2.75 g phenylalanine Sigma P-2126

- 6.75 g proline Sigma P-0380

- 3.40 g serine Sigma S-4500

- 2.25 g threonine Sigma T-8625

- 0.50 g tryptophane Sigma T-0254

- 3.25 g valine Sigma V-0500

- 1.75 g glycine Sigma G-7126

- 1.25 g methionine Sigma M-9625

Increase pH to 6.8 for proper dissolving of all amino acids

Store in –40°C freezer (stable for months)

**References:**

R. Otto, B. ten Brink, H. Veldkamp and W.N. Konings. 1983. The relation between growth rate and electrochemical proton gradient of  *Streptococcus cremoris. FEMS Microbiology Letter* **16**: 69-74

B. Poolman and W.N. Konings. 1988. Relation of growth of *Streptococcus lactis* and *Streptococcus cremoris* to amino acid transport. *Journal of Bacteriology* **170**: 700-707
